# One Hundred and Thirty Patients a Day!

**Published:** 1914-05-16

**Authors:** 


### THE PROFESSION OF MEDICINE.

### One Hundred and Thirty Patients a Day!

Just lately Manchester, not for the first time, has been one of the principal storm centres of the Insurance Act cyclonic disturbances. Furious attacks are being made by trades unionist and other representatives of approved societies upon the medical services rendered to insured persons by those practitioners who undertake such work. In Manchester there is, of course, no panel system worked upon the capitation-fee plan existing elsewhere, but a separate scheme of payments per attendance provided out of a pool which contains the whole allowance for medical benefit as received from the local Insurance Committee. Whether this Manchester system has had any share in causing the dissatisfaction which is being expressed by the insured, through their representatives, would require more local knowledge than we can pretend to possess. But certain figures which have been published by a member of the Manchester Insurance Committee render it quite possible that this is the case. This member, a Mr. Mellor, asserted that one doctor had been consulted in three separate months on 3,840, 3,906, 4,334 occasions. This comes out at 12,080 consultations in three months, or, roughly, 130 *per diem*, Sundays and holidays included. Mr. Mellor admitted, as, indeed, his experience no doubt compelled him to, that many of the complaints brought against the medical profession by Insurance Act patients are devoid of substantial foundation; but he was perfectly justified in adding that it is humanly impossible for any doctor to treat adequately such large numbers of patients as this particular practitioner had seen. One scarcely knows which to condemn most strongly, the degradation of the man who consents to do scamped work, or the Act of Parliament which makes it possible.

But there are other grounds for dissatisfaction in connection with the Insurance Act in Manchester. The chemists are up in arms about their remuneration, and all sorts of allegations are flying about concerning extravagant prescribing by the medical men. This difficulty is not peculiar to Manchester, however, but is endemic over a considerable portion of Britain. As elsewhere, too, the tuberculosis provisions of the Act are creating much dissatisfaction and grumbling. The *Manchester City News*, for example, devotes two columns to a review of the situation in this respect. Before the Insurance Act was passed the South Manchester Board of Guardians established a tuberculosis sanatorium at Abergele, which was just getting into working order when the Act threw upon the Manchester Corporation the duty of providing sanatorium accommodation for all insured persons within the city area who needed it. The South Manchester Guardians then transferred their sanatorium to the Corporation, stipulating, reasonably enough, that all phthisical cases from their own Union should be accepted at Abergele.

This stipulation, according to the article in question, is not being carried out. Advanced cases are not being accepted at Abergele at all, and even early cases, such as, beyond all others, ought to have sanatorium treatment, are kept waiting for long periods. At the end of April the Guardians had in their wards one hundred and sixty-three cases of phthisis alone, and further cases of non-pulmonary tuberculosis; and thirty-five cases of phthisis on outdoor relief. Even when the Corporation does provide residential treatment, it is asserted to be often very unsatisfactory. Thus at Baguley there is a "sanatorium" so-called, to which early cases are often sent. But the Local Government Board classify this institution as a "hospital" for advanced cases, many of which are admitted there.

If these contentions are correct, the Manchester Corporation is faced with responsibility for a very discreditable state of affairs. The excuse is, of course, lack of funds; and this is attributable to the breakdown of the finance of the Insurance Acts.

